# Relationships between recovery experiences and well-being among younger and older teachers

**DOI:** 10.1007/s00420-019-01475-8

**Published:** 2019-09-24

**Authors:** Anniina Virtanen, Jessica De Bloom, Ulla Kinnunen

**Affiliations:** 1 Faculty of Social Sciences (Psychology), Tampere University, Tampere, 33014 Finland; 2 University of Groningen, Groningen, The Netherlands

**Keywords:** Recovery from work, Recovery experiences, Aging, Teachers

## Abstract

**Purpose:**

The study had three aims. We investigated, first, how six recovery experiences (i.e., detachment, relaxation, control, mastery, meaning, and affiliation) during off-job time suggested by the DRAMMA model (Newman et al. in J Happiness Stud 15(3):555–578. 10.1007/s10902-013-9435-x, 2014) are related to well-being (i.e., vitality, life satisfaction, and work ability). Second, we examined how age related to these outcomes, and third, we investigated whether age moderated the relationships between recovery experiences and well-being outcomes.

**Methods:**

A sample of 909 Finnish teachers responded to an electronic questionnaire (78% women, average age 51 years). The data were analyzed with moderated hierarchical regression analyses.

**Results:**

Detachment from work, relaxation, control, and mastery were associated with higher vitality. Detachment, relaxation, meaning, and affiliation were related to higher life satisfaction. Older age was related to lower work ability, but not to vitality or life satisfaction. Older teachers benefited more from control and mastery during off-job time than did younger teachers in terms of vitality, whereas younger teachers benefited more from relaxation in terms of all well-being outcomes.

**Conclusions:**

Detachment, relaxation, control, mastery, meaning, and affiliation during off-job time were related to higher well-being, supporting the DRAMMA model. Age moderated the relationships between control, mastery, and relaxation and vitality and life satisfaction. The role of aging in recovery from work needs further research.

## Introduction

Recovery from work is an important factor in mitigating the relation between high job demands and ill-health (Geurts and Sonnentag 2006; Sonnentag et al. 2017). It refers to the process of alleviating strain symptoms caused by job demands (Sonnentag and Fritz 2015) and restoring employees’ energy and mental resources (Zijlstra and Sonnentag 2006). Aging is known to slow down the recovery process on a physiological level (Ilmarinen 1999), but the scientific evidence on the effects of aging on psychological recovery processes remains very limited. Due to the increasing number of aging people in the workforce, it is crucial to understand the challenges that older workers face and to generate strategies to support longer, healthy careers and prevent early retirement. Recovery from work can be assumed to help prolong working careers, because it is closely related to health and well-being (e.g., de Bloom et al. 2015; Fritz and Sonnentag 2006; Geurts and Sonnentag 2006). However, we do not have yet a clear understanding of psychological recovery processes among aging workers.

The target group of this study was teachers, who, according to several international studies, seem to be an especially stressed occupational group (e.g., Kinnunen et al. 1994; Kyriacou 2001; Salo 2002; Skaalvik and Skaalvik 2015). Teachers face job demands slightly different from those of other knowledge workers, although, for example, high workload is present in their daily working lives as it is in many other occupations. Typical teacher stressors mentioned in several studies include time pressure, students’ behavioral problems and low motivation, value conflicts, lack of recognition, lack of autonomy, conflicts with colleagues or parents, and the increasing use of technology in teaching (e.g., Betoret 2009; Fernet et al. 2012; Friedman 1995; Hakanen et al. 2006; Klassen and Chiu 2011; Kokkinos 2007; Skaalvik and Skaalvik 2009, 2011, 2017). Teachers also tend to spend a lot of time on work-related activities outside formal work hours (e.g., Garrick et al. 2018), which limits the time available for recovery from work. It is, therefore, important to find new ways to promote teachers’ recovery and specifically to identify experiences aiding recovery which have not received much attention in earlier research on aging employees or teachers.

The aim of this study is to contribute to recovery research in three ways. First, we focused on recovery from work among teachers, a highly loaded occupational group, whose recovery processes are under-examined. There is evidence showing that recovery is especially important when job stressors are high (Sonnentag 2018). Second, this is one of the first studies to investigate psychological recovery experiences (detachment, relaxation, control, mastery, meaning, and affiliation) suggested by the recently developed DRAMMA model (Newman et al. 2014) in the context of aging. Third, we examined whether age moderated the relationships between these recovery experiences and well-being. Thus, our study produces novel information about aging teachers’ recovery from work during off-job time.

### Recovery from work

Research so far has distinguished two complementary processes underlying recovery from work (De Bloom et al. 2010; Geurts and Sonnentag 2006; Sonnentag 2001). First, the passive mechanism suggests that recovery only occurs when people stop working and rest (Meijman and Mulder 1998). Low demands and disengagement from work are assumed to enable employees’ psychobiological systems to return to baseline levels (McEwen 1998; Sonnentag and Fritz 2015). Second, the active perspective of recovery highlights the importance of engagement in pleasant or challenging leisure activities (Geurts and Sonnentag 2006). The active perspective suggests that to recover from work stress, employees need to replenish threatened or lost resources (Hofboll 1989), and engage in activities which produce positive emotions and satisfy their basic needs for autonomy, relatedness, and competence (Fredrickson 2001; Ryan and Deci 2000). Summing up, recovery entails resting and detaching from work, but also building new resources and engaging in meaningful leisure activities.

Recovery can be elicited by certain subjective experiences, leisure-time activities, and physiological processes occurring during sleep (Sonnentag 2018). In this study, we focus on psychological recovery experiences underlying different leisure activities. Sonnentag and Fritz (2007) suggested a framework of four major recovery experiences: psychological detachment from work, relaxation, control, and mastery. Detachment refers to mental disengagement from work-related thoughts. Relaxation implies low levels of mental or physical activation and little physical or intellectual effort. Control refers to being able to decide on one’s leisure schedule and activities. Mastery encompasses learning opportunities and challenges, resulting in feelings of achievement and competence. Of these four experiences, detachment seems to be most consistently associated with positive changes in well-being (for reviews, see Sonnentag and Fritz 2015; Wendsche and Lohmann-Haislah 2017). Several studies have also demonstrated links between relaxation, control, mastery, and better well-being (for a meta-analysis, see Bennett et al. 2018).

Based on a meta-analysis of 363 articles within psychology and leisure sciences, Newman et al. (2014) added the experiences of meaning and affiliation to this list of recovery experiences in their DRAMMA model, which aims to explain how leisure activities relate to subjective well-being. They also replaced control with autonomy, which refers to feelings of decision latitude. Autonomy is also one of the basic psychological needs suggested in Self-Determination Theory (Ryan and Deci 2000). Autonomy closely resembles control, but is broader by emphasizing feelings of volition in general instead of merely having control over one’s leisure schedule (Newman et al. 2014). Meaningful leisure activities are a means by which individuals gain something valuable in their lives (Iwasaki 2008). Experiencing meaning in life is beneficial for well-being on both trait level (e.g., Hicks and King 2007; King et al. 2006) and state level (e.g., King et al. 2006; Machell et al. 2015; Thrash et al. 2010). Also, at day level, active search for meaning is related to improvements in well-being (Newman et al. 2018). This means that proactively engaging in activities that add meaning to one’s life is likely to improve well-being. Affiliation refers to feelings of belongingness with other people and the fulfillment of people’s innate need for relatedness (Ryan and Deci 2000). According to Newman et al. (2014), of all DRAMMA experiences, affiliation has the most support from multiple theoretical perspectives. In addition to fulfilling the basic psychological need for relatedness (Ryan and Deci 2000), social affiliation also fosters social support, which helps to mitigate against stressful events (Lakey and Orehek 2011). In this study, we investigated how these DRAMMA recovery experiences during leisure time (i.e., evenings after working hours) are related to three aspects of well-being: vitality, life satisfaction, and work ability.

Vitality and life satisfaction describe context-free well-being. Vitality refers to a positive feeling of aliveness and energy (Ryan and Frederick 1997). Since recovery from work allows employees to gain new internal resources such as energy and positive mood (Sonnentag and Fritz 2007), recovery experiences can be assumed to promote vitality. A meta-analysis by Bennett et al. (2018) showed that recovery experiences are related to higher vigor, which includes vitality and positive activated affect. Life satisfaction is a subjective global judgement of one’s quality of life (Diener et al. 1985) and a central component of subjective well-being (Diener et al. 2017). Previous studies show that recovery-related experiences are associated with higher life satisfaction (e.g., Sonnentag and Fritz 2007; Strauss-Blasche et al. 2002).

Work ability can be defined as the degree to which employees are mentally and physically capable of performing their current work role and of achieving a balance between a person’s resources and work demands (Ilmarinen et al. 1997; Tuomi et al. 1991). Work ability has its roots in health status (Ilmarinen 2009). Since recovery from work mitigates the relation between work stress and ill-health, and helps to build new resources (Geurts and Sonnentag 2006; Sonnentag et al. 2017), it can be presumed to promote work ability.

In addition, we examined whether age is related to these three well-being outcomes. Earlier research has shown that age is associated with decreases in work ability (e.g., Alavinia et al. 2009; Ilmarinen et al. 1997; Kinnunen and Nätti 2018). Some studies suggest that life satisfaction tends to reach a low point in mid-life but increases again after reaching retirement age (Blanchflower and Oswald 2008; Stone et al. 2010). This means that in our sample consisting of working people aged up to 68 years, aging may be associated with lower life satisfaction. Earlier studies suggest that although aging is generally related to higher affective well-being, this mostly applies to low-arousal positive states (e.g., relaxation, peace of mind), not more energized states like vitality (Kessler and Staudinger 2009; Scheibe and Zacher 2013). Some studies also show that aging may bring a shift in preference away from high-arousal positive emotions and towards low-arousal positive emotions (e.g., Scheibe et al. 2013). It could, therefore, be assumed that aging is either not related to vitality or related to lower vitality.

### Age, recovery, and emotion regulation

As stated previously, scientific evidence of the effects of age on psychological recovery processes remains limited so far. However, recovery processes are closely linked to emotion regulation (Parkinson and Totterdell 1999; Sonnentag and Fritz 2007; Sonnentag et al. 2017), and the motivation and competence for emotion regulation tend to change with age (Scheibe and Zacher 2013). Consequently, it can be assumed that aging may play a role in recovery from work.

It is important to note that the research streams of life-span development and organizational literature differ in terms of the definitions of “older” or “aging” people (Doerwald et al. 2016). In the life-span literature, age 60 or 65 is often used as a cut-off for when old age begins (Baltes and Smith 2003), whereas definitions of older workers correspond to the general operationalization of middle age, around 40–60 years (Doerwald et al. 2016). As this study is about teachers who are still working, we adhere to the definition for aging workers as it appears in the organizational literature (Doerwald et al. 2016).

The few existing studies about age and recovery have mostly focused on individuals’ own perceptions of their need for recovery, which seems to change during the life course. Two studies have shown that employees’ need for recovery after the working day increases linearly until the age of 55 and then stabilizes for the oldest workers approaching retirement age (Kiss et al. 2008; Mohren et al. 2010). Explanations for these findings can be found in three domains (Mohren et al. 2010). First, in the work environment, the process of downshifting may have been initiated, for example, in terms of a reduction in working hours. Second, differences in the family situation may account for varying levels of need for recovery: often, the oldest employees no longer have children living at home, which is likely to reduce work–family conflict and the demands of the family domain. Third, older employees may have developed better strategies for dealing with need for recovery due to their longer experience and expertise in their working careers (Silverstein 2008). Consequently, it is possible that older employees have better “recovery skills”. These skills relate to leisure crafting, which refers to the proactive pursuit of leisure activities targeted at goal setting, human connection, and personal development (Petrou and Bakker 2016).

The restoration of positive mood and energy are core functions of recovery from work, which supports the link between recovery and emotion regulation (Sonnentag and Fritz 2007). Research on emotion regulation has identified a range of strategies that individuals use to improve their mood, including both cognitive and behavioral strategies. Sonnentag and Fritz (2007) refer to the classification by Parkinson and Totterdell (1999), which proposes two main categories of emotion regulation: diversionary and engagement strategies. Diversionary strategies aim at avoiding a stressful situation or seeking distraction from it, whereas engagement strategies refer to confronting or accepting the stressful situation. According to Sonnentag and Fritz (2007), diversionary strategies are more relevant for work-stress recovery, because engagement strategies keep the individual cognitively occupied with the stressful situation, which makes recovery less likely. Diversionary strategies relate closely to three recovery experiences: detachment from work, relaxation, and mastery (Sonnentag and Fritz 2007). Higher age seems to be related to an increased preference to choose distraction (a less effortful, diversionary strategy) over reappraisal (an engagement strategy) when downregulating negative emotions (Scheibe et al. 2015).

Aging entails changes in emotion regulation motivation. Older adults seem to be more motivated to regulate emotions to optimize well-being, whereas younger adults are generally more focused on the achievement of goals (e.g., goals related to work and career development) (Carstensen 2006; Labouvie-Vief 2003). These changes are assumed to be driven by changes in future time perspective and cognitive abilities. In sum, higher age is associated with a higher motivation to avoid affective states that are negative and/or high in arousal (Scheibe and Zacher 2013). This is likely to have consequences for recovery, which focuses on dealing with job stress, a highly aroused negative state. It is possible that older employees, for example, have higher motivation to engage in detachment and relaxation during off-job time to distract from job stress.

Due to their greater life experience, older adults may also be more effective in implementing emotion regulation strategies and more competent in emotion regulation (Scheibe and Zacher 2013). Prominent life-span psychology theories, such as socioemotional selectivity theory (Carstensen 2006) and the model of selection, optimization, and compensation (Baltes and Baltes 1990), propose that aging triggers proactive behavior and is related to prioritizing emotional goals. These proactive behaviors, especially when they relate to emotion regulation and goal setting, may also be associated with recovery from work. Due to their long work and life experience, older workers may have a clearer understanding of what helps them to recover more successfully and make the most of their leisure time.

### The present study: research questions and hypotheses

In the present study, we sought answers to three research questions. First, we asked: How do recovery experiences of detachment, relaxation, control, mastery, meaning, and affiliation outside working hours relate to (a) vitality, (b) life satisfaction, and (c) work ability? Basing our examination on the DRAMMA model (Newman et al. 2014) and the existing research on recovery experiences (e.g., the meta-analysis by Bennett et al. 2018), we predict (H1) that all recovery experiences are related to higher well-being. Of the well-being outcomes, there is most evidence concerning the positive links to vitality.

Second, we asked: Is age related to vitality, life satisfaction, and work ability? We expect (H2) that age relates to lower work ability (e.g., Alavinia et al. 2009; Ilmarinen et al. 1997; Kinnunen and Nätti 2018), and likely also to lower life satisfaction (Blanchflower and Oswald 2008; Stone et al. 2010), and possibly to lower vitality (e.g., Kessler and Staudinger 2009; Scheibe and Zacher 2013), as discussed above.

Our third research question concerned the role of age in the relationship between recovery experiences and well-being outcomes. Thus, we asked: How does age moderate the relationship of recovery experiences and the outcomes described above? To the best of our knowledge, this issue has not yet been examined. Therefore, we did not formulate specific hypotheses regarding each recovery experience. In light of the existing literature about age-related changes in emotion regulation, we assume, for example, that detachment and relaxation may be more easily (i.e., with less effort) achieved by older teachers due to their greater motivation to avoid stress, which in turn is reflected in their higher levels of well-being. However, younger teachers may be in a greater need of detachment and relaxation due to their heavier family demands and, therefore, benefit more from these recovery experiences. All in all, concerning the last research question, our study can be considered explorative, although we expect (H3) to find moderator effects.

## Methods

### Participants and procedure

The participants of this study (*N* = 909) were teachers and school principals working in Finnish comprehensive or upper secondary schools. The sample was drawn in May 2017 from the register of the Trade Union of Education (OAJ). In Finland, around 95% of teachers are members of the trade union (OAJ 2015). The electronic questionnaire was sent to 3500 teachers all over the country by the union: to 1500 class teachers (teaching grades 1–6, i.e., pupils aged 7–12 years in comprehensive school), to 1500 subject teachers (teaching in either comprehensive school grades 7–9, i.e., pupils aged 13–15 years, or upper secondary school, i.e., pupils aged 16–18 years), and to 500 school principals. In the groups of class teachers and subject teachers, the questionnaire was sent to 500 teachers in three age groups: under 45 years, 45–55 years, and over 55 years. Due to the smaller total number of principals, this age division was not used in their group.

The response rate was 26% (*N *= 909). Among class teachers, it was 30% (*n* = 448), among subject teachers 28% (*n* = 321) and among principals only 21% (*n* = 140). The response rate was highest (37% among class teachers and 23% among subject teachers) among the middle-age group (45–55 years). The attrition analyses showed that the study participants were older (the share of teachers over 55 years old was 41.5% vs. 18.6%; *χ*^2^ (2) = 278.01, *p* < 0.001), more often women (83.4% vs. 77.6%; *χ*^2^ (1) = 14.65, *p* < 0.001), and subject teachers (47.1% vs. 35.6%; *χ*^2^ (1) = 12.66, *p* < 0.001) than teachers registered as members of the Trade Union of Education. The age difference is explained by the procedure through which the sample was drawn: as aging teachers were the target group of the study, the older age groups were given more weight than those under 45.

Of all the participants, 78% were women (86% of class teachers, 80% of subject teachers, but only 49% of the principals). The mean age of the participants was 51 years (SD = 9.76). Nearly all (99%) of the participants had a full-time job, and most (86%) also had a permanent employment contract. On average, participants worked 37.44 h per week (SD = 9.24). The majority (93%) of the participants worked in comprehensive schools (i.e., teaching students aged from 7 to 16 years). Most of the participants lived either with a partner (41%) or with a partner and at least one child (36%).

### Measures

#### Recovery experiences

Each recovery experience was measured with three items referring to one’s free time outside working hours. Psychological detachment (*α* = 0.82, e.g., “I forget about work”), relaxation (*α* = 0.80, e.g., “I kick back and relax”), control (*α* = 0.78, e.g. “I feel that I can decide for myself what to do”), and mastery (*α* = 0.68, e.g., “I seek out intellectual challenges”) were measured with items from the Recovery Experience Questionnaire (Sonnentag and Fritz 2007), which has been validated in Finland (Kinnunen et al. 2011). Meaning (*α* = 0.69, e.g., “I do things which are personally meaningful for me”) was measured with three items adapted from the Job Diagnostics Survey (Hackman and Oldham 1974). Affiliation (*α* = 0.77, e.g., “I really like the people I interact with”) was measured with three items from Basic Needs Satisfaction in General Scale (Johnston and Finney 2010), but one item (“There are not many people that I am close to”) was excluded from the analyses due to low Cronbach’s alpha (*α* = 0.44). All recovery experiences were rated on a scale from 1 (totally disagree) to 5 (totally agree). All Cronbach’s alphas reported for the scales of recovery experiences and other variables were calculated from our sample.

#### Moderator

Age as a moderator was used as a continuous variable in our analyses. Age was calculated from year of birth.

#### Well-being

Vitality was measured with four items from the scale by Ryan and Frederick (1997) (*α* = 0.89, e.g., “I felt alive and vital”). The items refer to feelings during the last month. The rating scale was from 1 (very rarely or never) to 5 (very often or always). Life satisfaction was measured with one item: “How satisfied do you generally feel about your life?” (e.g., Cheung and Lucas 2014) on a scale from 0 to 10. Work ability was measured with one item (“How would you rate your current ability to work?”) from the Work Ability Index (Tuomi et al. 1998). The item was rated on a scale from 1 to 10, where 1 refers to being totally incapable of working and 10 refers to one’s work ability at its best. It has been shown that this one-item measure accurately reflects the total work ability index (e.g., Jääskeläinen et al. 2016).

#### Controls

Several meta-analyses (e.g., Crawford et al. 2010; Nixon et al. 2011) indicate that individuals who are exposed to a higher level of job stressors report poorer well-being and poorer recovery experiences (Bennett et al. 2018). We, therefore, controlled for an important job stressor, workload, in our analyses. In addition, we controlled for one job resource, job autonomy, which is related to higher subjective well-being (e.g., Wheatley 2017). We also controlled for whether the participants had child(ren) living at home, because family situation may be related to recovery opportunities during off-job time. Finally, we controlled for leadership status, i.e., whether the participant was a school principal (= 1) or not (= 0), because managers may have heavier workload and, therefore, more problems with recovery than employees without leadership responsibility (e.g., Sonnentag and Fritz 2007).

Workload was measured with three items (*α* = 0.87, e.g., “How often does your job require you to work under time pressure?”) from the scale by Spector and Jex (1998). The items were rated on a scale from 1 (very rarely or never) to 5 (very often or always). Job autonomy was measured with six items (*α* = 0.78, e.g., “I can set my own work pace”) from QPSNordic-ADW (Pahkin et al. 2008). The items were rated on a scale from 1 (very rarely or never) to 5 (very often or always). The number of children living at home was elicited with one question: “How many children do you have who live in the same household with you?”. The answers to this question were recoded into a dichotomous variable (0, no children living at home; 1, at least one child living at home).

### Statistical analyses

First, we calculated means, standard deviations, and correlations between all study variables. Moderated hierarchical regression analyses (Aiken and West 1991) were used to test the direct effects of recovery experiences and age on three well-being indicators and the moderator effects between age and recovery experiences. We conducted hierarchical multiple regression analysis for each dependent variable using the following procedure: control variables (workload, job autonomy, having children living at home, and leadership status) were entered into the model at step 1, recovery experiences at step 2, age at step 3, and finally, the interaction terms of each recovery experience with age were entered at step 4 (6 interactions in total). Finally, we performed simple slope analyses to test the significance of the relationships among younger (1 SD below the mean age) and older (1 SD above the mean age) teachers. All recovery experiences, workload, job autonomy, and age were standardized in the regression analyses. All analyses were conducted in SPSS 24 software.

## Results

### Descriptive results

Means, standard deviations, and correlations between all the study variables are presented in Table 1. All recovery experiences correlated positively with vitality (0.18 ≤ *r* ≤ 0.42), life satisfaction (0.08 ≤ *r* ≤ 0.34), and work ability (0.10 ≤ *r* ≤ 0.26). Recovery experiences correlated positively with each other (0.09 ≤ *r *≤ 0.55), with the exception that the correlation between mastery and affiliation was not statistically significant. Well-being outcomes (vitality, life satisfaction, and work ability) were highly correlated with each other (0.48 ≤ *r* ≤ 0.55). However, none of these correlations between the six recovery experiences or the outcomes is over 0.85, which is considered a limit for concepts not being separate from each other (Hair et al. 2010). Age correlated negatively with work ability (*r* = − 0.08, *p* < 0.05), but was not significantly associated with vitality or life satisfaction. In addition, age correlated with higher detachment (*r* = 0.11, *p* < 0.01), relaxation (*r* = 0.10, *p* < 0.01), control (*r* = 0.07, *p* < 0.05), and mastery (*r* = 0.08, *p* < 0.05). Higher age was related to not having children living at home (*r* = − 0.24, *p* < 0.01). Workload correlated negatively with all well-being outcomes (− 0.15 ≤ *r* ≤ − 0.25), most strongly with vitality, and with recovery experiences (− 0.09 ≤ *r *≤ − 0.30), except for mastery and affiliation. Job autonomy was positively related to all outcomes (0.21 ≤* r* ≤ 0.33) and all recovery experiences (0.09 ≤ *r* ≤ 0.29). Having children at home correlated negatively with relaxation (*r *= − 0.17, *p* < 0.01), control (*r* = − 0.19, *p* < 0.01), and mastery (*r *= − 0.08, *p* < 0.05), but positively with affiliation (*r* = 0.14, *p *< 0.01). It was not significantly related to well-being outcomes. Leadership status correlated with higher age (*r *= 0.14, *p* < 0.001), workload (*r* = 0.11, *p* < 0.01), job autonomy (*r* = 0.31, *p* < 001), and vitality (*r* = − 13, *p* < 0.001).

**Table 1:** Means, standard deviations, and correlations between variables

Variable (range)	*M*	SD	1.	2.	3.	4.	5.	6.	7.	8.	9.	10.	11.	12.	13.	14.
1. Workload (1–5)	4.01	0.73	1													
2. Autonomy at work (1–5)	2.75	0.71	− 0.33***	1												
3. Having child(ren) living at home (0, no; 1, yes)	–	–	0.08*	− 0.03	1											
4. Leadership status (0, no; 1, yes)	–	–	0.11**	0.31***	− 0.01	1										
5. Detachment (1–5)	2.83	0.91	− 0.29***	0.24***	− 0.06	0.02	1									
6. Relaxation (1–5)	3.93	0.70	− 0.25***	0.20***	− 0.17**	0.01	0.54***	1								
7. Control (1–5)	3.93	0.74	− 0.30***	0.29***	− 0.19**	0.02	0.41***	0.65***	1							
8. Mastery (1–5)	3.34	0.73	− 0.04	0.11**	− 0.08*	0.05	0.15***	0.24***	0.20***	1						
9. Meaning (1–5)	4.36	0.57	− 0.09**	0.10**	− 0.04	− 0.02	0.23***	0.55***	0.43***	0.29***	1					
10. Affiliation (1–5)	4.58	0.51	− 0.06	0.09*	0.14**	0.00	0.09**	0.29***	0.33***	05	0.47***	1				
11. Age (18–68)	50.55	9.76	− 0.05	0.05	− 0.24**	0.14***	0.11**	0.10**	0.07*	0.08*	0.01	0.04	1			
12. Vitality (1–5)	3.25	0.83	− 0.25***	0.33***	− 0.01	0.13***	0.31***	0.42***	0.39***	0.27***	0.33***	0.18***	0.03	1		
13. Life satisfaction (0–10)	8.80	1.39	− 0.17***	0.21***	0.05	0.02	0.28***	0.34***	0.31***	0.08*	0.28***	0.23***	0.02	0.52***	1	
14. Work ability (1–10)	8.71	1.31	− 0.15***	0.27***	0.04	0.07	0.21***	0.26***	0.25***	0.11**	0.16***	0.10**	− 0.08*	0.48***	0.55***	1

*p < 0.05, **p < 0.01, ***p < 0.001

### Regression analyses: direct associations and interactions between age and recovery experiences

The results of regression analyses are presented in Table 2.

**Table 2:** Results of regression analyses, *β*’s from the last step of the model

Independent variables	Vitality		Life satisfaction		Work ability	
	Δ*R*^2^	*β*	Δ*R*^2^	*β*	Δ*R*^2^	*β*
Step 1	0.13***		0.07***		0.09***	
Workload		− 0.08*		− 0.04		− 0.04
Autonomy at work		0.16***		0.12**		0.20***
Child(ren) living at home		0.07*		0.11**		0.09*
Leadership status		0.08*		− 0.02		0.02
Step 2	0.16***		0.13***		0.06***	
Detachment		0.08*		0.10*		0.07
Relaxation		0.15**		0.10		0.09
Control		0.14**		0.13**		0.09
Mastery		0.14***		− 0.01		0.05
Meaning		0.08		0.11*		0.02
Affiliation		0.02		0.09*		0.02
Step 3	0.00		0.00		0.01*	
Age		− 0.01		0.01		− 0.08**
Step 4	0.01*		0.01		0.02*	
Age × detachment		0.03		− 0.02		0.08
Age × relaxation		− 0.14**		− 0.12*		− 0.19**
Age × control		0.09*		0.08		0.04
Age × mastery		0.08*		0.03		0.03
Age × meaning		0.03		− 0.03		0.09 (*p* = 0.058)
Age × affiliation		0.01		0.03		0.01
Total *R*^2^	0.30***		0.21***		0.17***	

*p < 0.05, **p < 0.01, ***p < 0.001

#### Vitality

**Fig. 1:**
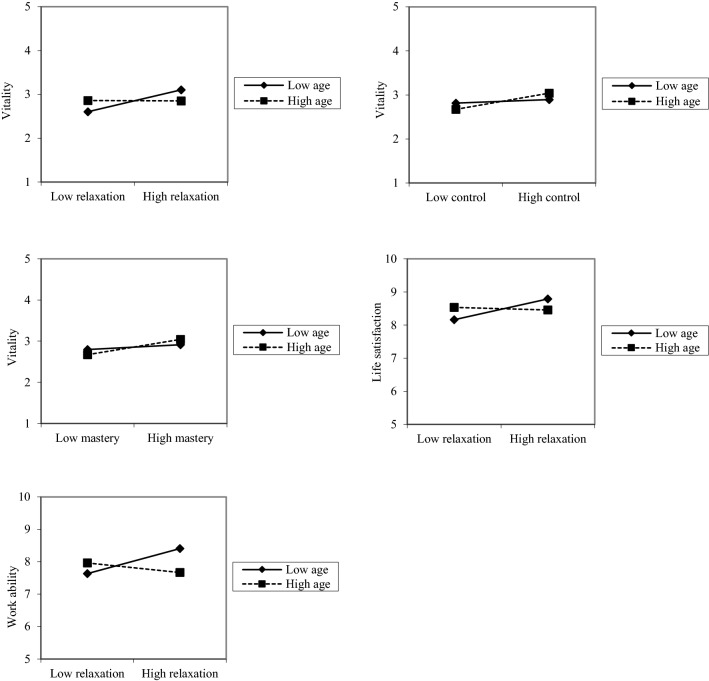
Interactions between age and recovery experiences

At step 1, job autonomy, having children living at home, and being a school principal were related to higher vitality. Controls explained 13% of the variance in vitality. At step 2, four recovery experiences predicted higher vitality: detachment, relaxation, autonomy, and mastery, with relaxation and mastery playing the major roles. Therefore, concerning vitality, H1 got partial support. Together, the recovery experiences explained 16% of the variance in vitality. Age did not predict vitality. In terms of this outcome, H2 was not supported. There were three statistically significant interactions between age and recovery experiences at step 4, giving partial support to H3. The graphical presentations of the interactions were derived using the unstandardized regression coefficients of the regression lines for teachers high (1 SD above the mean age, that is, over 60 years) and low (1 SD below the mean age, that is, under 40 years) on the moderator variable of age. As shown in Fig. 1, younger participants seemed to benefit more from relaxation experiences during off-job time than did older participants in terms of higher vitality (see Fig. 1a). However, older participants benefited more from control and mastery experiences than did younger ones (see Fig. 1b, c). The interactions added 1% to the explanation rate, and totally, the model explained 30% of vitality. The simple slope analyses (10) confirmed the age differences: the positive unstandardized regression coefficients (Bs) were higher and statistically significant for older teachers [control: *B* = 0.187, *p *< 0.001 (older) vs. *B* = 0.039, ns (younger); mastery: *B* = 0.183, *p *< 0.001 (older) vs. *B* = 0.057, ns (younger)], suggesting that older teachers benefit more from control and mastery than younger ones. The relationship between relaxation and vitality was positive in the younger age group (*B* = 0.245, *p *< 0.001), whereas the relationship was not significant in the older group (*B* = − 0.005, ns), suggesting that younger teachers benefit more from relaxation in terms of vitality.

#### Life satisfaction

At step 1, job autonomy and having children living at home were related to higher life satisfaction, explaining 7% of the variance in life satisfaction. At step 2, four recovery experiences (detachment, control, meaning, and affiliation) were related to higher life satisfaction, control playing the biggest role. Recovery experiences added 14% to the explanation rate. This gives support to H1. At step 3, age did not predict life satisfaction. Therefore, H2 was not supported in terms of life satisfaction. At step 4, one interaction effect turned out to be significant; H3 gained partial support, showing that younger participants benefited more from relaxation experiences than did older ones (see Fig. 1d). The simple slope analysis showed that in the younger age group, there was a significant positive relationship between relaxation and life satisfaction (*B* = 0.316, *p* < 0.01), whereas among the older group, the relationship was not significant (*B* = − 0.036, ns). This interaction added 1% to the explanation rate. In total, the model explained 21% of the variation in life satisfaction.

#### Work ability

At step 1, job autonomy and having children living at home were related to higher work ability, explaining 9% of the variation in work ability. In terms of work ability, H1 did not get support. At step 2, none of the recovery experiences predicted work ability significantly, but together they added 6% to the explanation rate. At step 3, greater age significantly predicted lower work ability, adding 1% to the explanation rate. This was in line with H2. At step 4, there was one significant interaction effect between age and relaxation, lending partial support to H3: again, younger participants seemed to benefit more from relaxation experiences than older participants (see Fig. 1e). The simple slope analysis showed that in the younger age group, there was a significant positive relationship between relaxation and work ability (*B* = 0.382, *p* < 0.001), whereas among the older group, this relationship was not significant (*B* = − 0.148, ns). Also, in terms of work ability, older participants seem to benefit slightly more from detachment, although this interaction was only marginally significant (*p* = 0.058). The interactions added 2% to the explanation rate, and in total, the model explained 17% of the variation in work ability.

## Discussion

The first aim of this study was to investigate how six recovery experiences—detachment, relaxation, control, mastery, meaning, and affiliation—during off-job time relate to vitality, life satisfaction, and work ability. Second, we examined whether age is related to these outcomes. Third, we investigated whether age moderated the relationship between recovery experiences and well-being outcomes.

### Main results

The results show that recovery experiences during off-job time are consistently related to context-free well-being, that is, feelings of positive energy, vitality, and a general cognitive evaluation of one’s life as a whole, life satisfaction. However, none of the recovery experiences predicted work ability, although at a correlational level, they had positive associations with this aspect of work-related well-being. Therefore, H1 got only partial support from the results. Empirical evidence on these links has also been presented (see Bennett et al. 2018, for a meta-analysis). Compared to vitality and life satisfaction, work ability is based more on physical health status (Ilmarinen 2009), which likely makes it more difficult to impact with leisure recovery experiences. All in all, the results of this study give support to the DRAMMA model (Newman et al. 2014): in addition to the four recovery experiences suggested by Sonnentag and Fritz (2007), leisure-time experiences of affiliation and meaning also promote well-being. Meaning was associated with both higher vitality and life satisfaction, whereas affiliation was only related to life satisfaction.

Age was not significantly related to vitality or life satisfaction, but, according to our expectations, higher age was related to lower work ability. This means that H2 also received partial support. Earlier research has also shown that work ability tends to decrease with age (e.g., Alavinia et al. 2009; Ilmarinen et al. 1997; Kinnunen and Nätti 2018). A few existing studies suggest that life satisfaction often reaches a low point in mid-life (which corresponds to 40–60-year old workers), whereas other hedonic aspects of well-being, like positive affect and happiness, are on an upward trajectory from youth to old age (Blanchflower and Oswald 2008; Stone et al. 2010). Our results did not show these age-related changes, which may be partly related to the fact that our study only included working people, while many earlier studies investigating age-related differences in psychological well-being have focused on older, retired individuals. In addition, we did not specifically study affective well-being (e.g., positive or negative affects), which tends to increase with age (e.g., Charles and Carstensen 2010; Scheibe and Carstensen 2010). Some studies have found no age-related differences in high-arousal positive affect (Kessler and Staudinger 2009). This is in line with our result, showing that age was not related to vitality.

All in all, older teachers seemed to recover better from work during off-job time than did their younger counterparts: age correlated with higher detachment, relaxation, control, and mastery. It is possible that due to their longer work and life experience, older teachers have learned more effective recovery skills and know what works best for them in relieving work-related stress. This is in line with earlier studies, suggesting that age is associated with higher competence in emotion regulation (Scheibe and Zacher 2013). Recovery skills can be linked to leisure crafting, the proactive pursuit of leisure activities targeted at addressing basic psychological needs (Petrou and Bakker 2016). The crafting perspective suggests that recovery from work is a process which can be actively shaped—it is not something which just automatically happens. Given that older teachers generally had higher levels of recovery experiences, it is an interesting question why they did not always benefit more from these than did younger teachers. In line with our third hypothesis (H3), we found that age moderated the relationship between some recovery experiences and well-being. Younger teachers seemed to benefit more than older teachers from relaxation experiences in terms of all three well-being outcomes. However, older teachers benefited more than younger teachers from control and mastery experiences during leisure time in terms of vitality.

There are several possible explanations for these moderator findings. First, age-related changes in family demands may play a role. Younger teachers more often have children living at home, which likely increases the demands of the family domain. Having high demands at both work and home, younger teachers may need relaxation more than do older teachers. Having children living at home and having relaxation experiences during off-job time were negatively correlated in our sample. The younger teachers may, therefore, have been in greater need of relaxation and, therefore, benefited more from it than did the older teachers. Second, the age-related differences in the relationship between leisure-time control and well-being may be explained by life-span theories. Socioemotional selectivity theory (Carstensen 2006) and dynamic integration theory (Labouvie-Vief 2003) suggest that older people prioritize emotional goals over achievement goals. It may be that leisure-time control is more important for older teachers than for younger ones, who are likely to focus more on work-related goals and raising children. Earlier research has also shown that striving for control, especially secondary control, such as changing one’s motives and goals, tends to increase with age (i.e., Heckhausen et al. 2010). Older teachers also seemed to benefit more from mastery experiences outside work than did younger ones. In terms of correlations, older teachers reported more mastery experiences than did younger ones, whereas having children living at home was related to fewer mastery experiences. It is probable that, due to differences in family situation, older teachers have more opportunities for these experiences (e.g., engaging in challenging hobbies) in their everyday lives. It is also possible that younger teachers have more mastery experiences at work (e.g., building up a career and learning new work-related skills), whereas older teachers start little by little to engage in downshifting and preparing for retirement. A diary study by Hewett et al. (2017) demonstrated that individuals benefit particularly from satisfaction of their need for competence in the home domain when it is not satisfied at work. This may be one reason why older teachers benefit more from mastery experiences during leisure time than do younger teachers, who may better satisfy their need for competence at work.

### Contributions and practical implications

The results of this study contribute to the literature in the following ways. First, our results lend further support to the recently developed DRAMMA model (Newman et al. 2014). In addition to the four recovery experiences suggested by Sonnentag and Fritz (2007), meaning and affiliation also seem to enhance well-being, which provides a more detailed perspective on recovery. Recovery may not only be a reaction to high job demands and experienced stress, but also preventive. For example, building personal resources through meaningful leisure activities and relatedness with other people may help employees to cope with upcoming stress. Affiliation or relatedness is often seen as a basic psychological need (Ryan and Deci 2000). It also fosters social support, which is consistently linked to good mental health (e.g., Lakey and Orehek 2011). In addition, several studies highlight the importance of meaning in life for psychological well-being (e.g., Machell et al. 2015; Newman et al. 2018; Thrash et al. 2010). Therefore, these two experiences are an important addition to the list of psychological experiences conducive to recovery from work.

Second, our results provide new insights into the role of aging in the psychological recovery processes, which has so far received limited attention in research. Our study showed that age played a role, as younger teachers benefited more from relaxation and older teachers benefited more from control and mastery during leisure time in terms of well-being. Third, we gained new information about recovery from work among teachers, who seem to suffer from high stress (e.g., Kinnunen et al. 1994; Kyriacou 2001; Salo 2002; Skaalvik and Skaalvik 2015). All six DRAMMA experiences were related to better well-being among teachers, which suggests that many different activities may be utilized to improve recovery.

In terms of practical implications, the results of this study suggest that to recover successfully from work, it is beneficial for teachers to engage in leisure activities that produce experiences of detachment, relaxation, control, mastery, meaning, and affiliation. Existing studies demonstrate that recovery from work can be supported with interventions such as relaxation techniques, recovery experience training, and promotion of physical activity (for a review, see Verbeek et al. 2018). In the future, the DRAMMA model and the findings of this study could be utilized to design more multidimensional recovery interventions addressing all six recovery experiences. In addition, recovery interventions targeted at specific occupational groups, like teachers, would be useful. It seems that among teachers, techniques related to distinguishing between work and private life and to reducing work-related rumination help people to detach and recover from work (see Ebert et al. 2015). Targeted interventions could take occupation-specific stressors into account and focus on specific strategies directed towards this occupational group. For example, one important stressor in teachers’ job is challenging interactions with pupils, so future interventions could possibly invent ways of mitigating the negative effects of these stressors on well-being and recovery.

Outside of interventions, employees can also proactively shape their leisure-time behaviors to meet their recovery-related needs. This is closely related to leisure crafting, a relatively new concept which deserves more attention in future studies. The findings regarding age-related changes in recovery processes suggest that different leisure activities may be beneficial for different age groups. Younger teachers may benefit more from engaging in relaxing activities, whereas older teachers especially would likely benefit from spending time on learning new things and developing their skills outside the work domain (e.g., engaging in challenging hobbies), because they benefited more from mastery experiences during off-job time. These age-related differences could be taken into account in designing recovery interventions. However, it has to be noted that personal preferences regarding specific activities likely also play a role in recovery processes. Moreover, it is possible that preferences for certain recovery experiences vary between individuals.

### Limitations and ideas for future research

One important limitation of this study is its cross-sectional nature: given that we only measured recovery experiences and well-being at one time-point, causal conclusions cannot be drawn. Longitudinal studies are needed to gain a more detailed picture of how and why aging impacts recovery from work. Within-person studies utilizing long time spans would also yield more information about how leisure experiences and recovery processes change during an individual’s life course. However, cross-sectional designs are recommended when conducting exploratory research such as ours (Spector 2019). In cross-sectional studies, generational effects may also play a role in explaining age-related differences: for example, research has previously addressed differences between generations in work and life values (Costanza and Finkelstein 2015; Zabel et al. 2017). It could be that for generations who put more emphasis on non-work values, recovery processes are more important (Sonnentag et al. 2017). Different age groups may also have different habits and preferences in terms of leisure-time activities, which may have an impact on recovery from work.

Another limitation relates to the sample of this study. The response rate was fairly low, and it is debatable whether, for example, the most stressed teachers did not have the energy to complete a relatively long questionnaire. In addition, the questionnaire was sent to the target group in May, which is an exceptionally busy time for teachers due to the end of the academic year. This study focused on teachers in Finland, which means that the results can be generalized to teachers only and that generalizing the results to teachers in different countries requires caution. Although teachers seem to have same job stressors worldwide, there are also certain differences between countries concerning, for example, the amount of technology used in teaching, students’ assessment practices, and the level of engagement required in extracurricular activities (OECD 2019). Future studies could pay more attention to the role of emotional job demands in teachers’ recovery processes, because the teacher’s job is emotionally demanding (e.g., Kokkinos 2007; Skaalvik and Skaalvik 2015, 2017). All in all, future research could examine recovery processes in different (aging) working populations in different countries around the globe.

In addition, our data were based on self-reports, and therefore, common method variance may affect the results. However, a number of factors in our study reduced the risk of common method bias (see Podsakoff et al. 2003). All our measures were derived from established questionnaires with good psychometric properties. In the questionnaires that we used the items for recovery experiences and outcomes had different scale anchors and the scale items were printed on different pages. Concerning interaction effects, according to Siemsen et al. (2010), common method variance actually deflates regression estimates of interaction effects, which means that these effects are not artificially created by common method variance. Although the use of self-reports has its limitations, it is indispensable when the focus of the study is on psychological experiences. Nevertheless, in future studies, it would be useful, for example, to combine physiological measurements like blood pressure or cortisol levels (which yield more detailed information about recovery processes on a physiological level) with the self-report data (for an overview of measurements in recovery research, see Sonnentag and Geurts 2009).

As we did not measure the participants’ personality or other individual characteristics in the questionnaire, we were unable to take into account their possible role in recovery and in predicting the well-being outcomes which we used. Many studies show that personality is related to health and well-being (e.g., Strickhouser et al. 2017; Sun et al. 2018), but little is known about the role of personality in recovery from work (Sonnentag et al. 2017). Future studies could pay more attention to this issue.

Although recovery from work has received a lot of scholarly attention, the role of specific leisure activities in supporting recovery could still be studied further. It is known that physical and social activities are usually conducive to recovery, but findings regarding most other types of leisure activities (e.g., passive activities, like watching TV) are inconsistent (e.g., Sonnentag 2001; Sonnentag et al. 2017). Although recovery experiences are presumed to underlie off-job activities (Sonnentag and Fritz 2007), little is known about which activities are linked to which experiences (e.g., Ragsdale and Beehr 2016). It is probably possible to get the same experiences from different activities and that the same activities may generate different experiences in different individuals (e.g., someone may find reading relaxing, whereas for someone else, it may produce mastery experiences). This issue deserves further investigation.

Age-related differences in recovery processes also need to be studied further. Further research is needed to find possible explanations for the interactions identified in this study. In addition, non-linear patterns could be taken into account in future studies. Earlier studies imply that the relationship between age and occupational well-being may be characterized by a U-shaped pattern, with younger and older employees experiencing better well-being than those in mid-career (see Zacher and Schmitt 2016). A wide age distribution is a prerequisite for such studies. In our sample, the mean age was relatively high and the number of young teachers was quite small. Also, several other relevant recovery outcomes, like burnout, could be taken into account. It would also be worth examining whether there are age-related differences in which leisure activities are beneficial in terms of recovery. In addition, personal preferences concerning recovery activities may change with age. This issue should be studied further. Previous studies have shown that motivational (e.g., extrinsic vs. intrinsic motivation) and affective attributes (e.g., enjoyment) associated with off-job activities play a decisive role in how specific activities support recovery from work (e.g., Sonnentag et al. 2017; Oerlemans et al. 2014; van Hooff and de Pater 2017; Waterman 2005).

## Conclusions

The results of this study suggest that six recovery experiences—detachment, relaxation, control, mastery, meaning, and affiliation—during off-job time are related to higher well-being among teachers. Older teachers seemed to benefit more from control and mastery experiences, whereas younger teachers seemed to benefit more than their older counterparts from relaxation. Possible practical implications include recovery interventions taking into account the role of age and occupation. Longitudinal studies are needed to learn more about the causal processes in recovery from work during an individual’s life course.
